# Analysis and Modeling of Innovations in the Global Microalgae Lipids Market

**DOI:** 10.3390/biotech11030037

**Published:** 2022-08-24

**Authors:** Natália Santana Carvalho, Luiggi Cavalcanti Pessôa, Kricelle Mosquera Deamici, Jania Betânia Alves da Silva, Fernanda Aleluia de Souza Parga, Carolina Oliveira de Souza, Pedro Paulo Lordelo Guimarães Tavares, Denilson de Jesus Assis

**Affiliations:** 1 Graduate Program in Chemical Engineering (PPEQ), Polytechnic School, Federal University of Bahia, Salvador 40210-630, Brazil; 2 Senai Cimatec University Center, Environment Department, Salvador 41650-010, Brazil; 3 GreenCoLab—Associação Oceano Verde, Algarve University, 8005-139 Faro, Portugal; 4 Center of Science and Technology, Mechanical Engineering Collegiate, Federal University of Recôncavo of Bahia, Cruz das Almas 44380-000, Brazil; 5 Undergraduate Program in Chemical Engineering, Salvador University, Salvador 41820-021, Brazil; 6 Graduate Program in Food Science (PGALI), College of Pharmacy, Federal University of Bahia, Salvador 40170-115, Brazil; 7 School of Exact and Technological Sciences, Salvador University, Salvador 41820-021, Brazil

**Keywords:** technological prospecting, patents, mathematical models

## Abstract

Microalgae lipids offer numerous advantages over those of plants and animals, enabling the sustainable commercialization of high value-added products in different markets. Although these markets are in a vertiginous annual expansion, technological life cycle modeling is a tool that has been rarely used for microalgae. Life cycle modeling is capable of assisting with decision-making based on data and is considered as a versatile model, usable in multiple software analyzing and diagnostic tasks. Modeling technological trends makes it possible to categorize the development level of the market and predict phase changes, reducing uncertainties and increasing investments. This study aims to fill this gap by performing a global analysis and modeling of microalgal lipid innovations. The Espacenet and Orbit platforms were used by crossing the keywords “microalgae”, “lipid*”, and the IPC code C12 (biochemistry and microbiology). Different sigmoid growth models were used in the present study. A successive repetition of the Chlorella genus category was found in the keyword clusters regarding extraction and separation of lipids. The life cycle S curve indicates a market starting at the maturity phase, where the BiDoseResp model stands out. The main countries and institutions at the technological forefront are shown, as well as potential technological domains for opening new markets.

## 1. Introduction

The growth of the population is increasing year by year, and it is estimated that the demand for the main sources to feed and maintain, such as energy, water and food, will increase until 2050 worldwide [1]. The use of waste for the development of products with high added value for human needs and the environment has become a worldwide trend, as it meets the challenges associated with the high costs of natural products, environmental impacts caused by waste disposal, and the energy crisis [2,3]. The high demand for lipids in the production of food, lubricants, detergents, biofuels, and nutritional supplements is conventionally derived from oilseed extraction and fossil fuels, which is one of the contributors responsible for the increase in the emission of polluting gases through the burning of organic matter [4]. However, this method may not satisfy the growing demand for this resource because of population growth, and, consequently, the decrease in available arable land and the increase in labor costs and environmental impacts [5,6].

An alternative to these limitations is the development of processes based on a circular economy, which can convert residual substrates into low-cost lipids [7]. To compete with plant-derived lipids in terms of cost, many microbial production technologies have been used in the cultivation of these organisms under specific conditions using agricultural residues as the carbon source. These microbial oils, also known as single-cell oils, have many advantages over plant-derived lipids, such as shorter life cycles, climate and season independence, no use of arable land, and ease of expansion [8].

Microalgae are simple microorganisms that stand out for their metabolic versatility and their ability to convert different compounds into high-value products, especially storage lipids [2,6]. Furthermore, they allow the integration of bioproduct synthesis and waste bioremediation. Pessôa et al. [9] carried out a comprehensive technological prospection involving microalgae biorefinery and effluent treatment, showing that this market, although promising, is still in its infancy, with technical challenges to be overcome. Rumin et al. [10] presented an overview of microalgae research with applications in potential markets until 2019, through the analysis of the main research concepts of the publications, the networks between them, the emerging concepts, and the annual production of articles. However, few studies have addressed technologies for obtaining and applying microalgal oils, encompassing their origins, evolution, market maturity, and socioeconomic context [11]. Thus, it is necessary to search for information on patent bases, as these documents provide information from across the world on technological innovations in the production and evolution of human inventive knowledge, providing data from various corporate and research sectors, which allows the identification of the level of technology maturity, inspiring actions, and providing opportunities for the perception of market needs.

Computational models are gaining attention with advanced software that may be employed for pathway prediction, data analysis, and optimization of bioproducts in microalgae cultivation [12,13]. Banerjee et al. [12] showed the importance of computational modeling and prediction of microalgae growth focused on improved lipid production. Mathematical models are usually included in the prediction behavior, reducing the cost and laboratory work time, and allowing prediction of the relation between microalgae growth with different cultivation conditions, linked to the formation of bioproducts.

Computational prediction makes it possible to reduce uncertainties and increase productivities, categorizing the level of the market and forecasting phase changes. Therefore, these tools are relevant in the long term since they could reduce the costs and the time involved in the process. Nevertheless, to the best of our knowledge, studies encompassing technological trend modeling are scarce, and the present study aims to fill this gap. In this context, the objective of this study is to carry out a global technological prospection based on the analysis of patent documents on lipids from microalgae biomass and its main applications through Espacenet and Orbit platforms. A comprehensive technological panorama is shown, covering the main depositors, companies that have been outstanding in the area, their collaboration networks, and emerging technologies. Moreover, the study aims to demonstrate and characterize the global maturity of this market, through a mathematical modeling profile of the technologies over the last 27 years. This study is expected to contribute information that subsidizes the development of new technologies and helps emerging companies in the area to minimize risks in decision-making for investments in research and development. 

## 2. Materials and Methods

Technology prospects were developed by searching patent documents in the European Patent Office (EPO) database, Espacenet (Munich, Germany), and Orbit (Paris, France). The search strategy consisted of combinations between the keywords “Microalgae” and “Lipid*”, in the title or abstract, and International Patent Classification Codes for the chemical and metallurgy areas, related to biochemistry, microbiology, enzymology, mutation, and genetic engineering (C12), and with specificity for microbiological processes (C12R). The combination was selected based on the specificity of the content and the number of documents to be analyzed. Espacenet data were retrieved in Excel (Redmond, United States of America) with the help of CSVed software and used to analyze the origin and annual evolution of documents, whereas Orbit was used to consult the predominant technological domains, the main depositors, and their collaboration networks. In addition, all abstracts were read, and the information contained in the documents made it possible to identify details of the innovations, such as microalgae genera and predominant applicators, characteristics of microbial lipids, and their applications.

The cumulative data on the number of documents over the years were adjusted to different sigmoid growth models (Boltziv, DoseResp, BiDoseResp, Logistic, Gompertz, and Richards) as they are widely used to adjust technology diffusion data and trace trends [14,15]. Technology diffusion is comprehensively accepted as tracing a sigmoidal curve that is similar to a biotic growth path. Therefore, those empirical growth models are useful for mimicking technology data [15].

**Figure 1:**
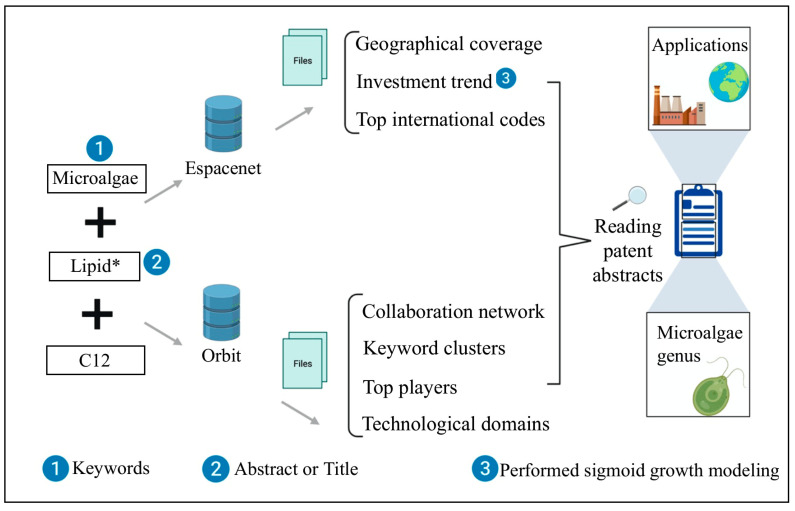
Illustrative scheme of the methodological steps performed. Image created with BioRender. * Truncation symbol representing the sequence of characters of any length.

OriginPro 8.1—OriginLab software was used to adjust the data. Non-linear models, including sigmoidal ones, are predefined in Origin. Figure 1 summarizes the methodology used in this study.

## 3. Results and Discussion

### 3.1. Table of Scope and Analysis of International Codes

Table 1 shows the results of the combinations of keywords and international classification codes. The search for “Microalgae”, “Lipid *”, and C12 code conditioned the retrieval of 274 documents, most likely to meet the scope of the research. It is also a feasible number for data refinement. The combination of the words “Microalgae” and “Lipid*” resulted in a greater number of documents (347). However, this strategy included documents dealing with technologies that go beyond the production and/or application of lipids of microalgal origin. In contrast, the use of the C12R code associated with keywords restricted the search for documents related to microbial processes, disregarding the subclasses of C12 related to the recovery of products, treatments, and applications.

**Figure 2:**
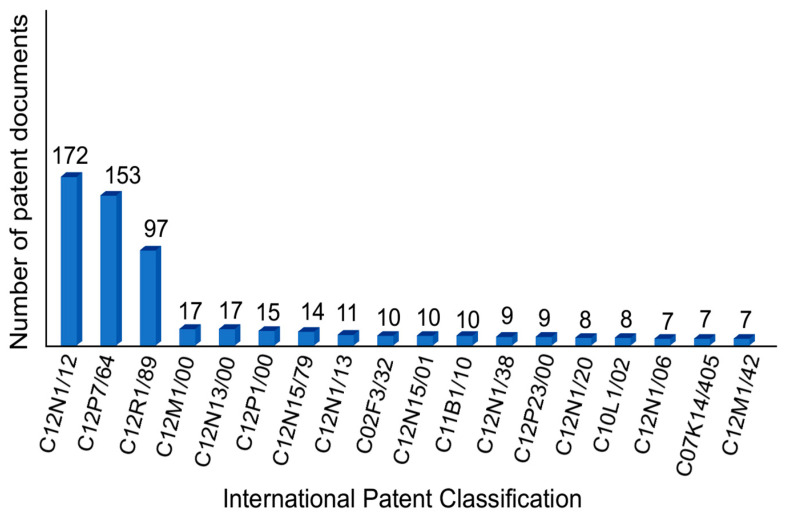
Distribution of patent documents by classification codes.

The distribution of patent documents by international classification codes (IPC) is shown in Figure 2. IPC organizes patents according to their technical fields through a system that classifies patents according to the topic. The data revealed that 63% of the recovered documents were applied to topics such as propagation processes, maintenance, preservation, and composition of unicellular algae, as well as preparations containing microorganisms (C12N1/12). Technologies involving the use of microalgae and the production of fats, oils, and waxes (C12P7/64) represented 56% of the recovered documents, whereas 35% dealt with processes using algae (C12R1/89). Thus, a significant portion of the retrieved documents were included in the research boundaries. The portion represented by codes with less than 10% participation in the documents covers technologies applied to the development of equipment for characterizing microorganisms and their use as a catalyst (C12M1/00), the treatment of microorganisms with electrical or magnetic energy (C12N13/00), and genetic mutations for performance improvements (C12N15/79). It should be noted that the number of codes exceeds the total number of documents because many documents fall under more than one classification.

### 3.2. Technological Domains and Main Products

**Figure 3:**
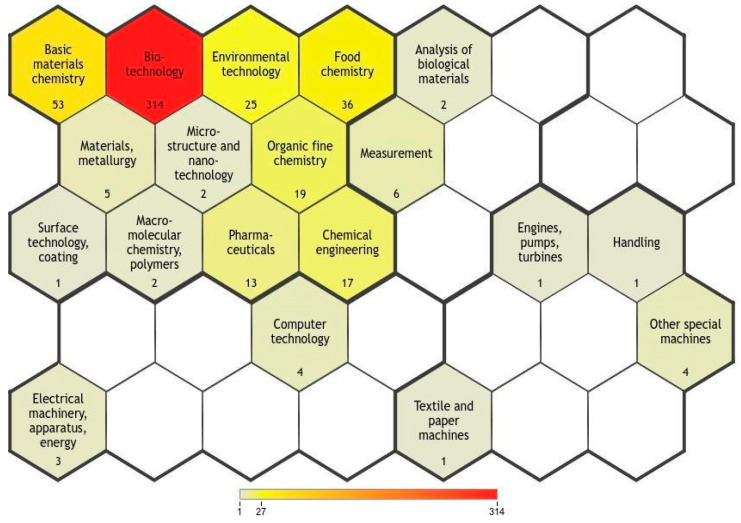
Technological domains involved in the prospected codes. The numbers show the quantity of documents in each technological domain.

The portfolio of technological domains can be extracted from the IPC codes themselves, which is a more visual way of analyzing the landscape of technological applications. Figure 3 illustrates the diversity of the economic sectors associated with microalgal biomass lipids. Note that more than one patent can be associated with more than one technological domain. Therefore, the total number counted was 314, that is, greater than the total prospected documents. The greater the color intensity, the more documents are associated with the technological domain. The numbers show the quantity of documents in each technological domain.

**Figure 4:**
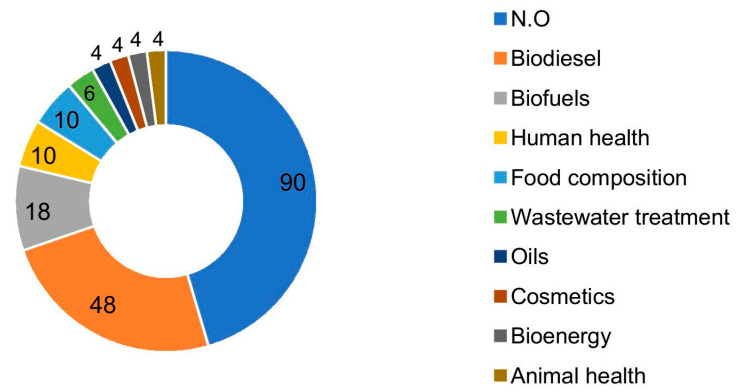
Number of documents by applications. “N.O” refers to unspecified.

Biotechnology (314), materials chemistry (54), and food chemistry (36) are the most prominent sectors; therefore, they are the most explored and prioritized market actions. Moreover, both computer technology (4) and microstructure and nanotechnology (2) are areas that present some interface with microalgal lipids but remain little explored. Notably, these are two key areas facing the new industrial revolution, the well-known Industry 4.0; therefore, they are developing markets in which startups can start exploring disruptive technologies. Among the applications reported in the literature, the most predominant was the production of biodiesel, present in 21.24% of the documents referring to production, followed by the production of biofuels, present in 7.96% of the documents (Figure 4).

Biodiesel is produced from vegetable oils and animal fat. In relation to microalgal biomass oil, conventional vegetable oils have two main disadvantages: first, they can be used for human consumption, which can generate an increase in the price of these oils used for food; and second, the high costs of biodiesel. Even if it has advantages over diesel oil, the high costs may limit production targets. Another disadvantage is that these raw materials can be grown in habitats that affect associated biological diversity. These concerns have increased the interest in the production of biofuels from non-food feedstocks. An alternative would be to use low-cost non-edible oils, such as used frying oils and animal fats; however, the quantities available are not sufficient to meet the current demand for biodiesel.

Thus, owing to these challenges, together with the high volatility of crude oil prices and the need to reduce greenhouse gas emissions, the use of microalgae for biodiesel production appears to be a viable alternative and arouses interest. Furthermore, compared to other crops, microalgae have a high oil content; therefore, in the long term, industrial microalgal use will offer greater opportunities and a rapid growth rate [16,17]. From an environmental perspective, the third generation of biofuels has been strongly encouraged in the world, especially from the primer on the water-energy-food nexus [9,18]. Algal fuels, unlike those derived from petroleum, are sulfur-free and have a low ecological footprint, reducing the impacts of climate change and the greenhouse effect [19,20]. From a marketing perspective, the evidence in Figure 4 suggests that biofuels from microalgal lipids have been one of the main applications. Biodiesel (48), biofuels (18), and bioenergy (4) patents have been added, totaling 70, against 90 unidentified patents. The Global Market Insight report highlights that algae-based biofuels can replace petroleum-based fuels and are among the fastest growing sectors worldwide [21].

The analysis carried out by the website’s analysts is that the leading companies in the global algal oil market should invest in Research and Development (R&D) during the period 2022–2028, improving the production of algal oil and increasing their collaborations, mainly in biofuels for the transport sector. This report also mentions that the main leading companies are: Cargill, Lonza Group, Seaweed Energy Solutions, TerraVia Holdings, Royal DSM, Cyanotech, Algix, Alltech, Diversified Energy, and DIC. However, as will be explained later, the evidence suggests that none of these companies have played a leading role in the deposition of technologies or cooperation with research centers.

Although this number is significant for biofuels and illustrates a technological race for the industrialization of algal biofuels, there is still energy infeasibility associated with the cultivation, harvesting, and processing of algae. Thus, for fossil fuel substitution to be fully considered, a biorefinery approach must be considered [22,23].

It should be noted that human health follows in the sequence, after biofuels. According to Global Market Insights [24], the EPA/DHA (omega-3) market will grow by 2026 at an annual rate of 7.2%, while the algal oil market for infant formula is estimated to grow by more than 8.5% per year until 2026 [25]. The report points out that changing consumer lifestyles and increasing urbanization are key factors for the growth of these markets.

### 3.3. Temporal Evolution of Documents and Mathematical Modeling of the Technological Life Cycle

**Figure 5:**
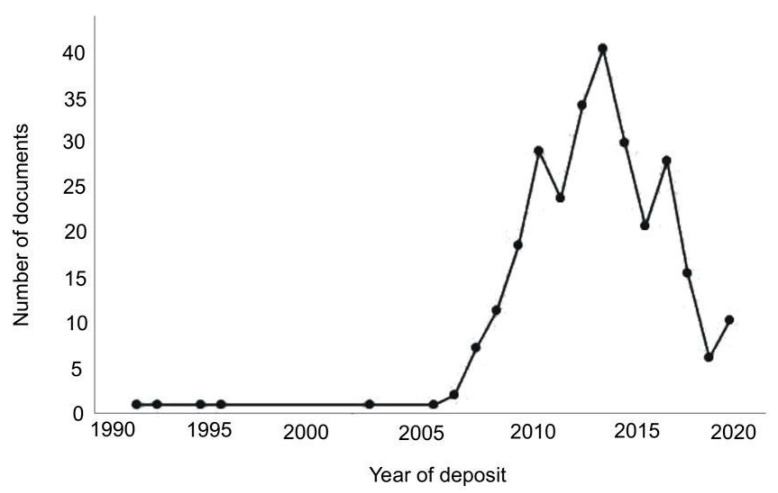
Number of documents by years.

The graph in Figure 5 represents the annual evolution of the number of patents filed regarding the use of microalgal biomass in the production of oils between 1993 and 2020. The historical context is a major factor for this growth in the number of patent applications. Since the oil crisis of the 1970s, there has been considerable interest in the search for alternative energy sources [26]. In the early 1970s, the oil crisis severely affected the French economy, and the French government sought to restructure its energy policy to reduce its dependence on imported oil supplies [27].

The data showed that the first patent application in the area was requested in 1993 by a French state-owned energy research company. Its innovation dealt with the development of a process responsible for the selective microalgal production of polyunsaturated lipids for energy application (EP0554162A1). The 1990s were also marked by the emergence of international agreements, such as the Kyoto Protocol, which was created in 1997 with a commitment to reduce the greenhouse effect and pollutants that cause global warming.

This context has further highlighted the importance of sustainable production and, consequently, the interest of industries, universities, and research institutes in developing technologies aimed at sustainable development [28]. These and other related events have corroborated the increasing number of patent documents in recent years. Since 2008, the number of patents requested has increased, which may have occurred as a result of greater interest in the biotechnological potential of microalgae because of their wide biodiversity and the large number of substances they can synthesize [29].

The apex of the global financial crisis occurred in 2008, together with the oil crisis, both of which encouraged the development of alternative energy sources [30]. In 2010, there was a significant increase in patents compared with previous years. In this period, scientific studies have demonstrated the potential of microalgae cultivation to produce oils for biodiesel, compared to the production from traditional land cultures [31]. Subsequently, worldwide interest has aroused in developing innovative methods to optimize the production and properties of microalgae oils. During the period evaluated, the largest number of patent filings occurred in 2014, with a total of 39 patent documents, which may be related to greater financial investment by investor countries [32,33].

In addition, 2013 was marked by the Conference of the Parties (COP-19), which may have been an important factor in the growth in the number of patents in 2014, as a new agreement to reduce emissions was established. The agreement includes measures that presuppose climate damage, and the financing of developing countries that already suffer from climate change due to the activities of developed countries. In other words, COP-19 sought to get governments to define projects to reduce the emission of gases that aggravate the greenhouse effect [34,35].

From 2017 onwards, there was a sharp decrease in the number of registered documents. This behavior is expected and is associated with confidential documents, which correspond to 18 months from the filing date, guaranteed by the Industrial Property Law to the depositor [36]. 

The life cycle of microalgae lipid technologies can be measured by calculating the parameters from fitted sigmoid empirical models. According to Cantú and Pedroza [37], the curve generated by the annual accumulation of patents allows us to classify the development stage of a technology as emerging, growing, mature, or declining. Thus, the models can characterize and justify which growth phase the prospected technologies are in by assisting start-ups, R&D decision makers, and investors. The statistical data of adjustment to the growth models, shown in Table 2, revealed that, except for the Richards model, the cumulative patent data showed good adjustments to the other models tested (R^2^ > 0.999). Among the well-fitted and significant models for the F test (*p* < 0.01), the BiDoseResp model showed the highest correspondence with the data (99.96%) and the highest F-value (15,665.72), indicating that its mean squares were greater than those of the residue; therefore, more significant than others.

The BiDoseResp model, represented in Equation (1), is characterized by double sigmoid behavior, according to its growth rate.

(1) $$PD=A1+\left(A2-A1\right)\left[\frac{p}{1+10^{\left(t1-t\right)h1}}+\frac{1-p}{1+10^{\left(t2-t\right)h2}}\right]$$

**Figure 6:**
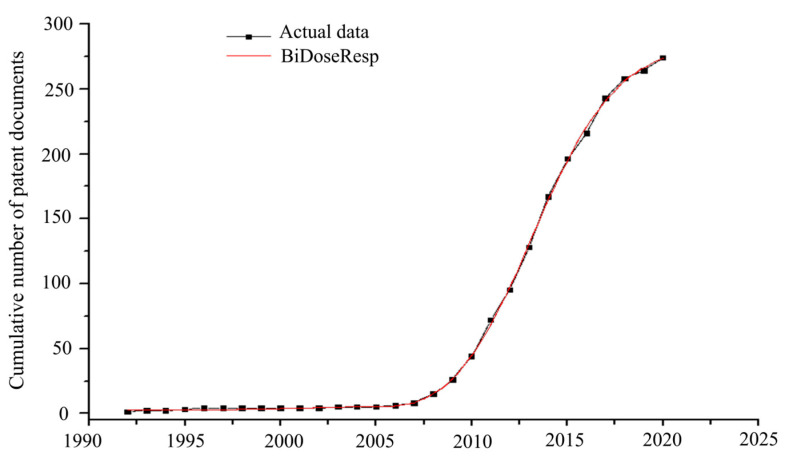
The technological trend of microalgae lipids.

*PD* is the cumulative number of patent documents; *A*1 and *A*2 are asymptotic values at the bottom and top of the curve, respectively; *p* is the ratio of the two segments; *h*1 and *h*2 are the slope coefficients of the first and second segments, respectively; *t*1 and *t*2 are the characteristic times of the two segments, respectively. The technological trend of microalgae lipids adjusted to the BiDoseResp model is shown in Figure 6.

Adjustment of the technological trajectory of microalgae lipids to the BiDoseResp model (Figure 6) resulted in mathematical data (Table 3), which allowed the identification of the technology development stages.

The results showed an unclear difference between the phases (segments) of the technology in question. The first phase started in 1993 and showed a tendency to decline in approximately 2010, while the second was in progress. In addition, the ratio between the segments showed a value < 0 (*p* = −0.062). This indicates that the number of documents on the border of the two segments was closer to the initial value, and the amplitude of the first segment in the curve was smaller than that of the second [38].

The values of *t*1 and *t*2, shown in Table 2, indicate that the first and second phases of the technology reached state-of-the-art development stages in 2005 and 2013, respectively. At this stage, the patent application rate (h) is at its maximum. Thus, interest in innovations involving microalgae lipids showed a higher growth rate in the first phase (*h*1 = 0.389), 12 years after the first patent application. In the second phase, the growth rate was twice as low (*h*2 = 0.193) and reached 20 years after the beginning of the first phase.

**Figure 7:**
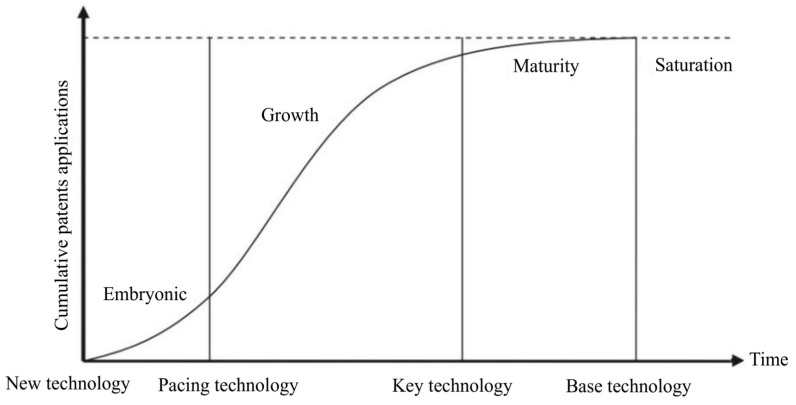
Typical S-curve of the life cycle of technologies. Source: Adapted from Taylor and Taylor [39].

A comparison of the curves shown in Figure 5 and Figure 6 indicates that the development of technologies related to microalgae lipids is in the early stage of maturity, according to the technology life cycle map shown in Figure 7. 

Studies have pointed out that technologies in the maturity stage tend to become key technologies, characterized by their integration into products or processes and high levels of competitive impact [40,41]. Innovations involving microalgae lipids are at an opportune moment to be commercially explored to meet current socio-economic and environmental emergencies. It is noteworthy, however, that, as microalgae lipids cover a broad spectrum of applications and technological domains previously discussed, applications for human health seem to be much more advanced compared to biofuels.

### 3.4. Geographic Coverage, Main Investors, and Cooperation Networks

**Figure 8:**
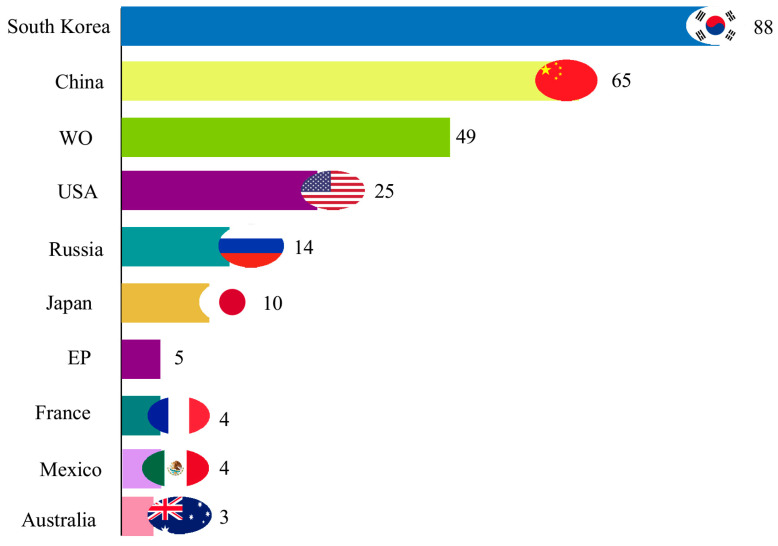
Distribution of patent filings by country. EP: European Patent Office; WO: World Intellectual Property Organization. Created with flourish.studio [43].

The data presented in Figure 8 reveal that South Korea presented the highest number of deposits related to oils accumulated by microalgae, approximately 32.12% of the total documents recovered. Next was China, followed by the World Intellectual Property Organization (WIPO) and the United States of America. Other countries presented 17.15% of the total number of patents filed. Historically, East Asian countries, such as South Korea, China, and Japan, have sought to diversify their energy sources to reduce dependence on fossil oil and meet the global trend in sustainable production [42].

These countries have introduced technological and political initiatives for the development of biofuels and assert themselves as a potential source of ethanol and biodiesel, due to the pressure generated by their domestic needs and commitment to reducing greenhouse gas emissions [42]. In addition, these nations have incentives for R&D, which contribute to an increase in technological products. In South Korea, the Ministry of Land, Transport, and Maritime Affairs invested USD 200 million in research to develop a methodology for producing biofuel using algae and microalgae [44].

In 2016, China had a 60% increase in its foreign investment in renewable energy, reaching a record of USD 32 billion, which includes the investment abroad of 11 new businesses worth more than USD 1 billion each. In 2015, China invested more than USD 100 billion in clean energy, which represents more than double the US investment and was a stimulus for the considerable generation of jobs. In 2017, of the 8.1 million renewable energy jobs that existed globally, 3.5 million were in China, and less than a million were in the United States.

With this, China is a strong candidate to become a world leader in this sector, and with the growth of clean energy in the domestic market, the country has built fundamental economies of scale for the export of clean energy products and services at a competitive cost [45].

The analysis of the information contained in the document summaries made it possible to identify details of the innovations, such as applicants, microalgae genera, predominant objectives, production data, products obtained, energy/carbon sources used, and applications. The data showed that the teaching and research institutions that deposited the most documents related to microalgal biomass oils were located in South Korea. In the early 1990s, Korean companies sought to innovate and compete in the market. The following were among the measures adopted: the transfer, through formal mechanisms, of foreign technologies; hiring of highly qualified labor from other countries; investment in R&D, especially in the educational sector and biotechnology; and government investments in research centers and universities [46,47]. These actions were essential for establishing an intellectual property protection system in the country.

**Figure 9:**
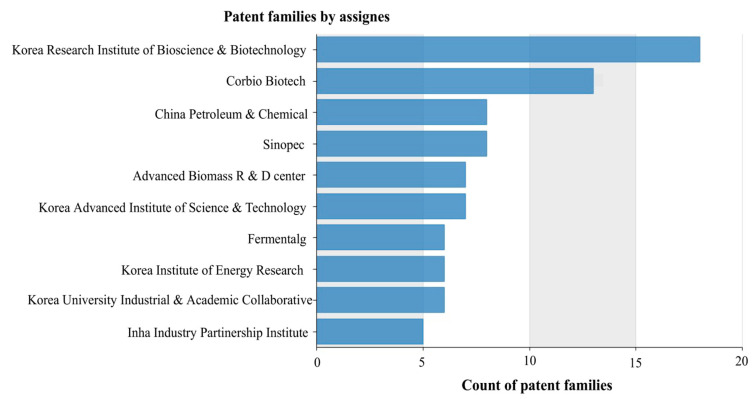
Top ten applicants for documents in relation to microalgae lipids.

The data in Figure 9 show the distribution of documents by applicants, considering the ten main institutions framed in the following sectors: companies, universities, research institutes, and cooperation agreements. The main research institute was the Korea Research Institute of Bioscience and Biotechnology, and the main company was Corbion Biotech. Considering its short existence, emphasis can be placed on the Advanced Biomass R&D Center (ABC). It was founded in 2010, to promote the development of microalgae-based biorefinery technologies with significant financial support (approximately USD 100 million over nine years) from the Korean Ministry of Science, ICT, and Future Planning (MSIP), leading to the establishment of a comprehensive and diversified innovation ecosystem encompassing more than 25 research groups in the specialties of psychology, molecular biology, chemistry, and chemical engineering.

To better utilize microalgal biomass to produce biofuels and biomaterials at a commercial level, new strains of microalgae with prominent features and energy-efficient technologies for mass cultivation, harvesting, and conversion are being intensively developed [48]. The data in Figure 9 show the distribution of documents by applicants framed in the following sectors: universities, research centers, companies, and cooperation between inventor institutions.

Notably, the Dutch Corbion Biotech is among the companies that deposit the most patents. Its portfolio of sustainable products stands out for its omega-3 from algae, as an alternative to fish oil, supporting healthy diets, and reducing pressure on marine resources without affecting the carbon footprint [49]. With six patents in the area, the French company Fermentalg is also a company that sells vegan omega-3 in a sustainable way through strains of *Schizochytrium* sp. According to the company’s website, the products are obtained in the form of natural triglycerides, without any physicochemical concentration steps, through refining processes without complex chemistry [50].

**Figure 10:**
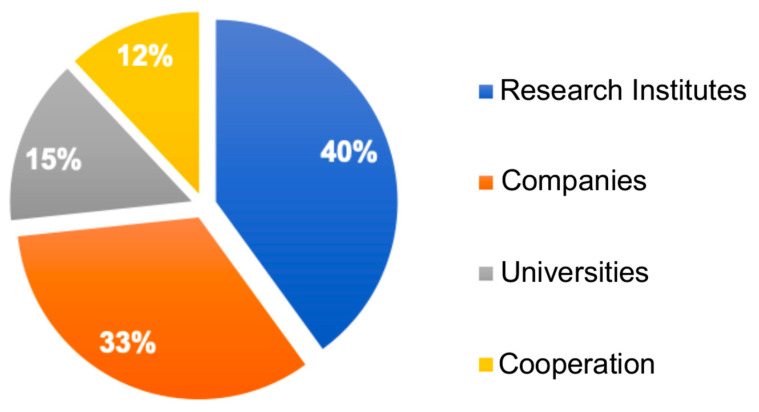
Number of documents by institutions.

Figure 9 illustrates that six of the top ten institutions are located in South Korea. South Korea has reached high levels in terms of technological and economic development. This progress is based on premises, such as investment in education and training of human resources, use of reverse engineering to favor assimilation capacity on the part of researchers, training for industrialization, and export incentives. South Korea’s efforts seek to conquer international markets, since a significant part of the Korean economy revolves around exports [46,47,51]. The data in Figure 10 show the distribution of documents categorized by institution according to the data extracted from Orbit for the 30 largest depositors.

Research institutes and companies have deposited more patent documents related to oils of microalgal origin, totaling 73% of the deposits. This information converges with the modeling of the patent documents presented in Figure 6 and Figure 7, showing that the life cycle is at the beginning of the maturation phase; that is, in the third phase. After reaching this stage, commercial risks tend to be reduced, and technology transfer takes place; therefore, the contribution of universities tends to be smaller than that of research centers and companies [52].

**Figure 11:**
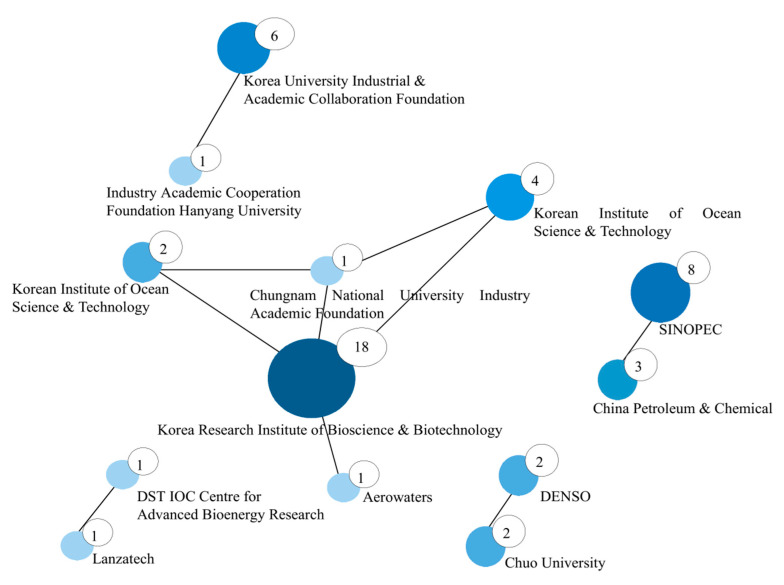
Interaction networks between the main depositors and their respective institutions. The numbers represent the quantity of interactions of the main depositors.

Figure 11 illustrates the cooperation networks between the portfolios of applicants. This figure is in line with Figure 8, Figure 9 and Figure 10, which illustrate the greater role of South Korea and its respective institutions. The larger the diameter of the circle and the thickness of the line, the greater the interaction between them. The numbers represent the quantity of interactions of the main depositors.

Two pieces of evidence can be inferred from this analysis: the prominence of South Korea in terms of the synergy between institutions and universities, and how these cooperations tend to be restricted to the national level. Institutions are mostly cooperating within their national innovation ecosystems. In the coming years, this successful model among institutions will inspire other countries, with expectations to reach beyond national borders.

### 3.5. Microalgae and Keyword Clusters

**Figure 12:**
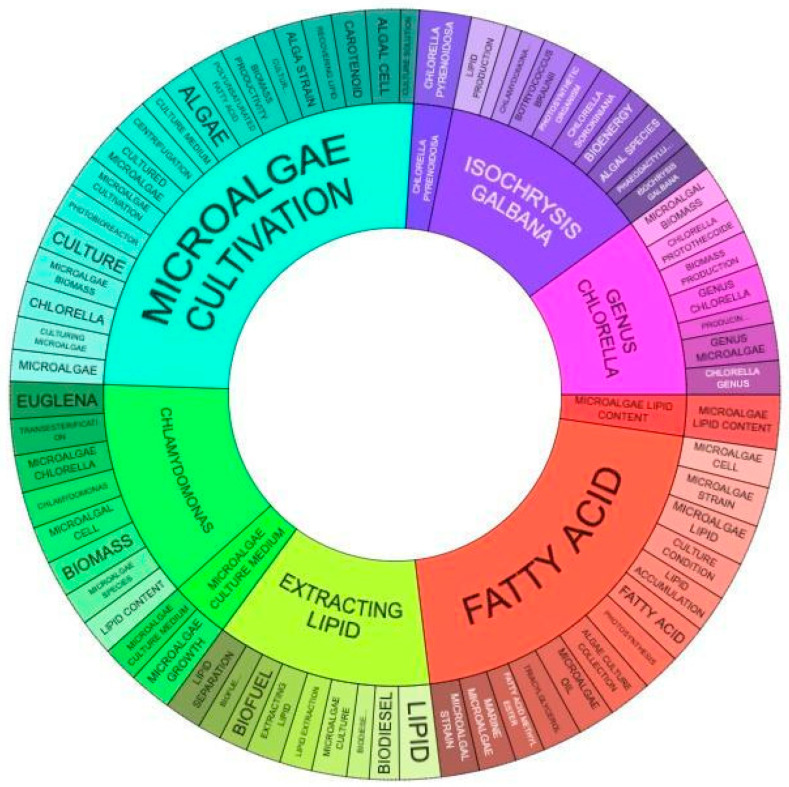
Clusters of the most repeated keywords in the document portfolio.

Figure 12 presents six clusters of keywords that are most repeated within the prospected patent portfolio: (i) microalgae cultivation, (ii) fatty acid, (iii) extracting lipid, (iv) genus *Chlorella,* (v) *Isochrysis galbana*, and (vi) *Chlamydomonas.* As word size refers to the number of repetitions, some key information can be extracted from each cluster. For example, regarding the first, microalgae cultivation, polyunsaturated acids seem to stand out, as well as carotenoids. For the second, the red cluster, marine microalgae species seem to be preferred, as well as patents aimed at optimizing lipid accumulation. For the third cluster, the majority presence of biofuels is noted, especially biodiesel, which is in line with Figure 4 regarding technological applications.

To a lesser extent, there is the presence of documents aimed at the extraction and separation of lipids, likely related to methods. Regarding the *Chlorella* genus, there is a successive repetition of this genus category. This correlates with the literature, given that studies have shown that this genus has metabolic characteristics that favor the synthesis of lipids, with the ability to accumulate lipids at 28–32% of its dry mass [53,54]. According to Feng et al. [55], the culture of *Chlorella vulgaris* reached the highest lipid content, 42%, in cultivation with wastewater treatment. The fifth cluster, in turn, portrays the minority presence of the *Isochrysis* genus, also focused on bioenergy.

The last cluster, of the genus *Chlamydomonas*, has the words “biomass”, “*Euglena*” and “lipid accumulation” highlighted. In a study by Fan and Zheng [56] the highest lipid content was 35.8% for this microalga in a combination of heterotrophic culture and multiple stresses (saline and light). Regarding the reading of patent documents, a similarity with Figure 12 was noted, as the genus *Chlorella* was mentioned in 14% of the documents, while *Chlamydomonas* was in second place, mentioned in 11%. On the other hand, 64% of the documents did not specify the microalgae genus; however, two genera appeared with tiny percentages of 5% (*Phaeodactylum)* and 3% (*Tetraselmis)*. This difference can be attributed to the greater number of words covered by Orbit in comparison to Espacenet, given the greater scope of the search, so these two genres have not been identified by the clusters.

## 4. Conclusions

This study sought to analyze and model the technological trends encompassing microalgal lipids over the last 27 years. The analysis of the temporal evolution of innovations shows a technological race with the first growth peak in 2006, while the S-curve modeling suggests the beginning of the market maturity phase for the clustering of technologies prospected. To the best of our knowledge, modeling the technological life cycle of microalgae is still scarce in the literature and can be further explored in future studies. For example, knowledge of the individual life cycles for each third-generation biofuel, mainly biodiesel and bio-jet fuel, will be instrumental. Priority research can help companies promote competitive advantages and reduce risks in R&D managers’ decision making.

A limitation of this study was the use of the C12 code, which restricted the search to biochemistry, microbiology, mutation, and genetic engineering. As the “A” classification is designated for human health, future studies may complement the search by working with subclassifications of that group.

The experience of South Korea’s synergistic institutional arrangement can be mimicked by other countries to advance the technological frontiers of microalgae biotechnology more rapidly. Disruptive technologies in this sector hold promise because, as they are platforms for a variety of sustainable products with high added value, a circular economy based on algae benefits not only countries but, above all, the future of the next generation.

## Figures and Tables

**Table 1:** Search strategy based on patent documents.

Keywords		Codes		Total
Microalgae	Lipid *	C12 ^1^	C12R ^2^	
X		X		4027
X			X	1472
X	X			347
X	X		X	110
X	X	X		274

^1^ Biochemistry, microbiology, enzymology, mutation and genetic engineering. ^2^ Processes involving microorganisms. * Truncation symbol representing the sequence of characters of any length. X: Symbol to indicate the codes and keywords used in the combinations.

**Table 2:** Adjustment of the accumulated number of documents to sigmoid growth models.

Model	F-Value	*p*-Value	R^2^
BoltzIV	11,042.05	<0.01	0.9990
DoseResp	14,390.94	<0.01	0.9992
BiDoseResp	15,665.72	<0.01	0.9996
Logistic	14,490.49	<0.01	0.9992
Gompertz	7888.21	<0.01	0.9981
Richards	42.11	<0.01	0.7767

**Table 3:** BiDoseResp model parameters for technological trend of microalgae lipids.

	*A*1	*A*2	*t*1	*t*2	*h*1	*h*2	*p*
Value	2.873	287.162	2005.952	2013.171	0.389	0.193	−0.062
Standard error	0.617	3.201	0.187	0.138	0.205	0.01	0.035
R^2^	0.9996						

## Data Availability

Not applicable.
